# ENO1 as a Biomarker of Breast Cancer Progression and Metastasis: A Bioinformatic Approach Using Available Databases

**DOI:** 10.1177/11782234241285648

**Published:** 2024-10-19

**Authors:** Athina Giannoudis, Alistair Heath, Vijay Sharma

**Affiliations:** 1 School of Dentistry, Institute of Life Course and Medical Sciences, University of Liverpool, Liverpool, UK; 2 Department of Cellular Pathology, Liverpool Clinical Laboratories, Royal Liverpool Hospital, Liverpool University Hospitals NHS Foundation Trust, Liverpool, UKK; 3 Institute of Systems, Molecular and Integrative Biology, Department of Molecular and Clinical Cancer Medicine, University of Liverpool, Liverpool, UK

**Keywords:** ENO1, breast cancer, biomarker, bioinformatic analysis, progression and metastasis

## Abstract

**Background:**

Metabolic reprogramming is one of the hallmarks of cancer, and in breast cancer (BC), several metabolic enzymes are overexpressed and overactivated. One of these, Enolase 1 (ENO1), catalyses glycolysis and is involved in the regulation of multiple signalling pathways.

**Objectives:**

This study aimed to evaluate in silico the prognostic and predictive effects of ENO1 expression in BC.

**Design:**

This is a bioinformatic in silico analysis.

**Methods:**

Using available online platforms (Kaplan–Meier [KM] plotter, receiver operating characteristic curve [ROC] plotter, cBioPortal, Genotype-2-Outcome [G-2-O], MethSurv, and Tumour–Immune System Interaction Database [TISIDB]), we performed a bioinformatic in silico analysis to establish the prognostic and predictive effects related to ENO1 expression in BC. A network analysis was performed using the Oncomine platform, and signalling, epigenetic, and immune regulation pathways were explored.

**Results:**

ENO1 was overexpressed in all the analysed Oncomine, epigenetic, and immune pathways in triple-negative, but not in hormone receptor–positive BCs. In human epidermal growth factor receptor 2 (HER2)-positive BCs, ENO1 expression showed a mixed profile. Analysis on disease progression and histological types showed ENO1 overexpression in ductal in situ and invasive carcinoma, in high-grade tumours followed by advanced or metastasis and was linked to worse survival. High ENO1 expression was also associated with relapse-free, distant metastasis-free and overall survival, irrespectively of treatment and was mainly related to basal subtype.

**Conclusion:**

ENO1 overexpression recruits a range of signalling pathways during disease progression conferring a worse prognosis and can be potentially used as a biomarker of disease progression and therapeutic target, particularly in triple-negative and in ductal invasive carcinoma.

## Background

Breast cancer (BC) is the most prevalent malignancy in women, and despite the advancements in its diagnosis and management, it is still one of the leading causes of cancer-related death in women.^1,2^ Its incidence and mortality have been reported to be 46.8% and 13.6%, respectively.^2,3^ BC is a very heterogeneous disease that can be classified by its histological subtype, its receptor status, or its molecular phenotype.^4,5^ The mainstay of oncological management of BC includes endocrine therapy, human epidermal growth factor receptor 2 (HER2)-targeted therapy, and chemotherapy.^6^ However, in addition to its heterogeneity, the presence of additional cell types, such as stromal and immune cells within the tumour microenvironment, makes more difficult the management of the disease.^7^ There is therefore a need to identify further targets that can be used as biomarkers of resistance and disease progression and be potentially used as therapeutic options.

Alterations in energy metabolism by cancer cells can promote tumourigenesis and it is well established that metabolic reprogramming is one of the hallmarks of cancer cells.^8,9^ There is a growing interest in exploring metabolic pathways for biomarkers and novel therapeutic targets. Glucose metabolism in cancer has received a lot of attention, mainly through the expression of glucose transporters (GLUT1/3), pyruvate dehydrogenase kinase (PDK), lactate dehydrogenase (LDH), hexokinases (HK1/2), phosphofructokinase (PFK), and its regulation by oncogenes, tumour suppressors, and transcription factors.^10^ In addition, various signalling pathways, such as Notch, phosphoinositide-3-kinase (PI3K), phosphatase and tensin homolog deleted (PTEN), mammalian target of rapamycin (mTOR), and mitogen-activated protein kinase (MAPK) also interact with metabolic reprogramming.^8-10^ During the last decade, despite the emerging of transporter and metabolic enzyme inhibitors, the efficiency of targeting glucose metabolism has proved challenging and there is a clinical need to identify and explore more promising targets. One of these targets is Enolase 1 (ENO1), a glycolytic enzyme that primarily catalyses the conversion of 2-phosphoglyceric acid to phosphor-enol-pyruvic acid during glycolysis.^11,12^ It is a multifunctional protein, ubiquitously expressed in most human tissues under normal and pathophysiological conditions, and is found overexpressed in many cancers.^11-13^

ENO1 mRNA and protein overexpression have been linked to disease progression and worse clinical outcomes in lung, breast, pancreas, glioma, head and neck, and colorectal cancers.^13-19^ In several cancers, such as gastric, pancreatic, prostate, and breast, in addition to worse outcomes, it has been associated to treatment resistance and in particular, chemoresistance.^20-22^ In BC, it has been shown in vitro that silencing ENO1 inhibits the proliferation, migration, and invasion of BC cells,^23,24^ and in a xenograft mouse model, inhibition of ENO1 expression increased tolerance to hypoxia in tumour cells, showing also slow reduced tumour size, cell growth, and increased apoptosis.^25^ A recent, single-cell transcriptomic profiling of BC patients identified higher ENO1 expression in the aggressive basal (triple-negative) subtype, compared with hormone- and HER2-positive subtypes, and this overexpression was linked to worse relapse-free survival (RFS).^26^ They also showed that depletion of ENO1 in triple-negative BC cell lines halted cell proliferation, colony formation, and tumour growths (3D-organoids) and increased cell death suggesting that ENO1 could be used as a therapeutic target in this aggressive subtype.^26^ However, a more comprehensive data analysis will allow us to better characterise the role of ENO1 in BC progression and its potential use as a targeted therapeutic agent.

The aim of this analysis is to evaluate the predictive and prognostic value of ENO1 as a biomarker of BC progression and as a therapeutic target using a range of online bioinformatics tools. Network analysis on the BC patient cohorts available on the Oncomine platform^27^ will allow us to comprehensively characterise how ENO1 clusters globally with genes involved in all known cancer hallmarks, epigenetic, and immune pathways.

## Methods

### Kaplan–Meier and receiver operating characteristic curve plotter analysis

The predictive and prognostic effect of ENO1 expression at mRNA level for all available BC Kaplan–Meier (KM)-plotter cohorts was assessed using the KM plotter (www.kmplot.com) tool.^28^ The BC cohorts were further stratified by treatment and by molecular subtype (PAM50) using the standard setting as previously described.^28^ Briefly, the expression of ENO1 was divided into high and low groups by splitting the mRNA expression level at the median values using the autoselect best cut-off recommended setting.^28^ KM survival analysis was performed to assess the effect on progression-free (PFS), distant metastasis-free (DMFS), and overall survival (OS). RFS is defined as the time from initial diagnosis to first recurrence of the disease. It is commonly used in trials as a surrogate marker of OS as it requires less follow-up to get this measure, and the information is available more quickly. DMFS is defined as the time from initial diagnosis to distant site (distant lymph nodes, lung, liver, brain), and OS is the time from initial diagnosis to death from any cause. For all the survival analysis, a log-rank *P*-value < .005 was considered significant.

The effect of ENO1 on treatment response was assessed using the receiver operating characteristic curve (ROC) and calculating the area under the curve (AUC) using the ROC-plotter tool (www.rocplot.org).^29^ The ROC-plotter uses a large transcriptomic database that includes treatment data and expression data from 3104 BC samples and provides an easily accessed resource to mine the database and rank biomarker candidates.^29^ ROC analysis was performed for complete pathological response (CPR) (n = 1775; responders: n = 639, non-responders: n = 1136) and 5-year RFS (n = 1329; responders: n = 978, non-responders: n = 351) as short- and long-term outcomes.^29^

### Genomic alterations analysis

Alterations in the ENO1 genome were assessed using the cBioPortal tool^30,31^ and the prognostic effect of these alterations was assessed by KM survival analysis using the KM-plotter and the Genotype-2-Outcome (G-2-O) tools.^32^

### Oncomine network analysis

Network analysis was performed using the Oncomine platform as previously described using the built-in molecular concepts.^27,33^ The signalling pathways for the cancer hallmarks, including immune regulation, were based on the NanoString concepts,^34^ and the epigenetic signalling pathways were obtained from the EpiFactor website.^35^ The assessment of the prognostic effect of methylation of the ENO1 gene was assessed using the MethSurv tool^36^ by KM analysis.

Briefly, ENO1 was investigated across the molecular concepts to identify clustering with different signalling pathways. Clustering of the signalling pathways with ENO1 was taken as significant at a *P* < .01 and any odds ratio (OR) (Supplementary file 1). The tool specified whether the clustering occurred in the context of over- or underexpression and specified the patient subgroup in which the clustering occurred. Subgroups identified in the platform included (1) subgroups related to stage, recurrence, and survival outcome and (2) subgroups related to the histological subtype and receptor status of the tumour.

### ENO1 and tumour–immune system interactions

The relationship between ENO1 and the immune response in BC was accessed using the Tumour–Immune System Interaction Database (TISIDB; http://cis.hku.hk/TISIDB/index.php).^37^ TISIDB integrates a variety of heterogeneous data sources and can help researchers find new immunotherapy targets, forecasting the effectiveness of immunotherapy. Our data were obtained using the TISIDB portal^37^ for all available lymphocytes, chemokines, and immunomodulators, and Spearman’s correlation (*r*) was used to determine the correlation of ENO1 expression and immune markers (*r* < 0.2 no correlation, 0.2–0.4 low, 0.4–0.6 moderate, 0.6–0.8 strong, and 0.8–1.0 very strong).

## Results

### ENO1 expression at mRNA level and correlation to survival

**Figure 1.**
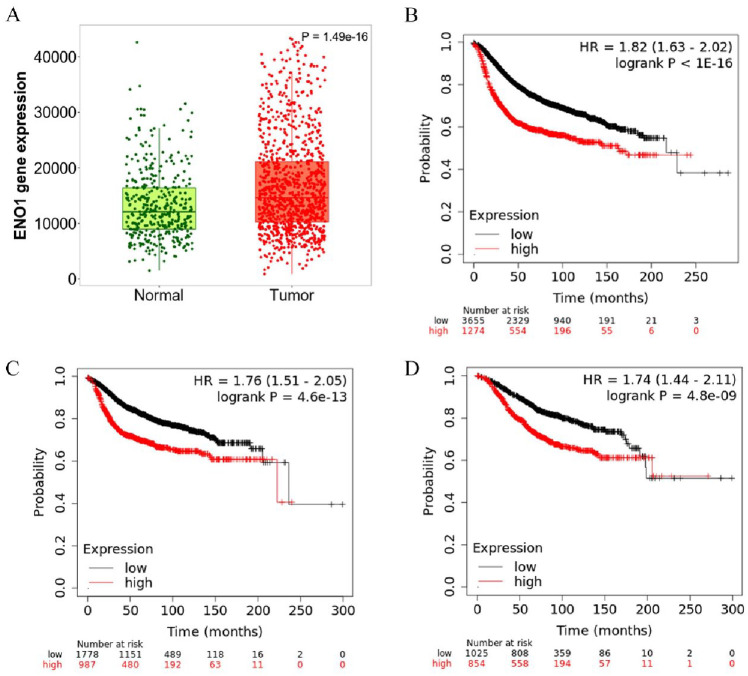
ENO1 mRNA expression in BC. ENO1 is overexpressed at mRNA level in (A) BCs in comparison to normal tissue (Tumour: n = 1097, Normal: n = 403; *P* = 1.49 × 10^−16^) and this expression is significantly higher in basal (n = 172) and HER2 (n = 73) subtypes compared with luminal A (n = 508), luminal B (n = 191), and normal (n = 137) (Kruskal–Wallis H-test *P* = 4.79 × 10^−84^). (B to D) High expression of ENO1 in BC patients correlates with a decreased (C) RFS, (D) DMFS, and (E) OS (*P* < .001 for all comparisons).

**Figure 2.**
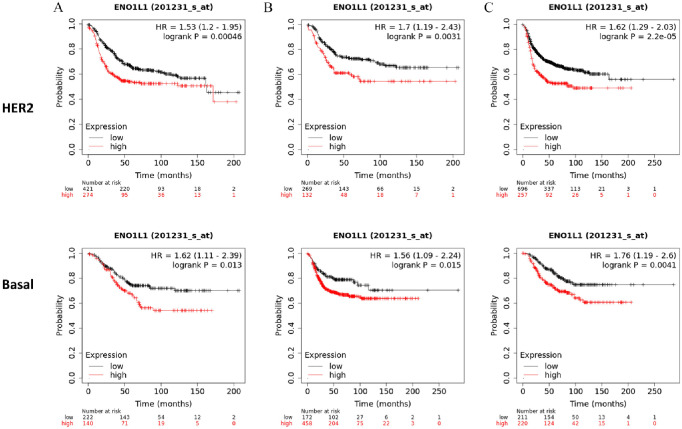
BC survival and ENO1 expression. Patient stratification by HER2 and triple-negative/basal molecular subtype showed a decreased (A) RFS, (B) DMFS, and (C) OS in HER2-positive and basal BC subtypes (*P* < .05 for all comparisons).

**Figure 3.**
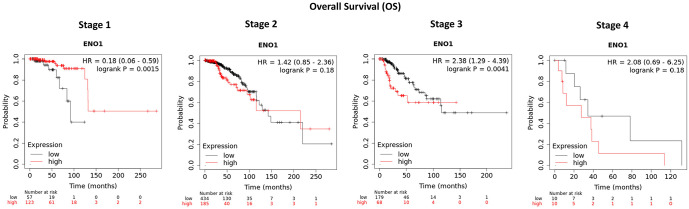
ENO1 mRNA expression by BC stage. ENO1 overexpression has a good prognostic effect in Stage 1 disease (*P* = .0015), no effect in Stage 2 (*P* = .18), and a poor prognostic effect in more advanced Stage 3 disease (*P* = .0041). The significance is not present in Stage 4 due to the very low number of samples (n = 10 in both arms).

ENO1 is overexpressed at mRNA level in BCs in comparison to normal tissue (Tumour: n = 1097, Normal: n = 403; *P* = 1.49 × 10^−16^; Figure 1A) and its expression is significantly higher at PAM50 basal (n = 172) and HER2 (n = 73) subtypes compared with luminal A (n = 508), luminal B (n = 191), and normal (n = 137) (Kruskal–Wallis H-test *P* = 4.79 × 10^−84^; Figure 1B). Overexpression of ENO1 is a poor predictive marker as identified by the KM-plotter tool (Figure 1C to E) using the Affymetrix ID: 201231_s_at (ENO1, ENO1L1, Myc promoter-binding protein-1 [MBP-1]). High expression of ENO1 in BC patients correlates with a decreased RFS, DMFS, and OS (*P* < .001 for all comparisons; Figure 1C to E, respectively). Stratifying the patients by treatment status (systemically treated and systemically untreated) including all types of treatment showed that high ENO1 expression predicted a poor survival outcome in all patients irrespectively of treatment (Supplementary Figure 1A to C). Patient stratification by molecular subtype showed a decreased RFS, DMFS, and OS in HER2-positive and basal BC subtypes (*P* < .05 for all comparisons; Figure 2A to C). No significant association was observed with luminal A, whereas luminal B subtype correlated only to decreased RFS (*P* = .0022; Supplementary Figure 2). When the effects of the gene were examined by stage, interestingly ENO1 overexpression has a good prognostic effect in Stage 1 disease and a poor prognostic effect in more advanced Stage 3 disease (Figure 3). At protein level, there was no correlation of ENO1 expression and survival (*P* = .095; Supplementary Figure 3).

**Figure 4.**
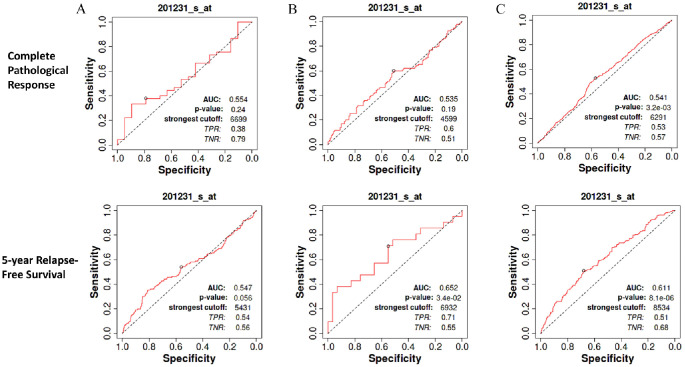
ENO1 expression and response to therapy. (A) There was no correlation between ENO1 and endocrine therapy in RFS or CPR between responders (R) and non-responders (NR) (R: n = 45, NR: n = 19 for CPR and R: n = 771, NR: n = 136 for RFS). (B) a moderate significant correlation (R: n = 29, NR: n = 21; AUC = 0.652, *P* = .034) was observed in RFS in patients treated with anti-HER2 therapy but there was no correlation to CPR (R: n = 95, NR: n = 122). (C) In chemotherapy-treated patients, there was moderate significant correlation with both CPR (R: n = 532, NR: n = 1100; AUC = 0.541, *P* = 0.032) and 5-year RFS (R: n = 256, NR: n = 220; AUC = 0.611, *P* < .0001).

We also carried out an analysis using the ROC-plotter tool^29^ to investigate whether ENO1 was a biomarker of sensitivity or resistance to endocrine therapy, HER2-directed therapy, or chemotherapy in BCs. Analysis was performed both in terms of CPR and 5-year RFS between responders (R) and non-responders (NR). There was no correlation between ENO1 and endocrine therapy (R: n = 45, NR: n = 19 for CPR and R: n = 771, NR: n = 136 for RFS; AUC < 0.55, *P* < .05), whereas a moderate correlation (R: n = 29, NR: n = 21; AUC = 0.652, *P* = .034) was observed in RFS in patients treated with anti-HER2 therapy (Figure 4A and B respectively). However, in chemotherapy-treated patients (Figure 4C), there was a moderate correlation with both CPR (R: n = 532, NR: n = 1100; AUC = 0.541, *P* = .032) and 5-year RFS (R: n = 256, NR: n = 220; AUC = 0.611, *P* < .0001). The data highlight the positive correlation between ENO1 expression and chemotherapy, and suggest that patients with high ENO1 expression are or may develop resistance to chemotherapy.

### Genomic alterations in ENO1

**Figure 5.**
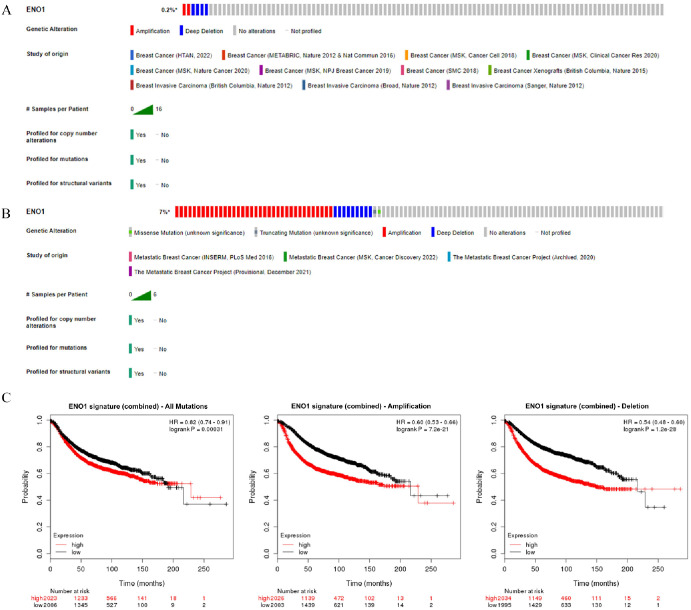
ENO1 genomic alterations and survival. Genomic alterations in the ENO1 gene were detected in (A) 0.2% of the primary BCs cohorts, mainly involving CNAs (gene amplifications and deletions) and in (B) 7% of the metastatic BC cohorts, mainly gene amplifications, deletions missense, and truncating mutations. (C) The metagene signatures associated with ENO1 alterations predicted a poor clinical outcome for all types of alterations (*P* < .001). BC cohorts and number of cases from cBioPortal are shown in Supplementary Table 1.

Data from cBioPortal^30,31^ showed that genomic alterations in the ENO1 gene were detected in 0.2% of BCs and they mainly involved copy number alterations (CNAs; gene amplifications and deletions). The BC cohorts and the data are presented in Figure 5A and Supplementary Table 1. Interestingly, analysis of the metastatic BC cohorts (Figure 5B) showed 7% genomic alterations (gene amplifications, deletions missense, and truncating mutations) potentially highlighting that alterations in the ENO1 gene are acquired during treatment and could be related to disease progression and metastasis. The metagene signatures associated with ENO1 alterations predicted a poor clinical outcome for all types of alterations (mutations, amplification, and deletion; Figure 5C).

### Oncomine network analysis

**Figure 6.**
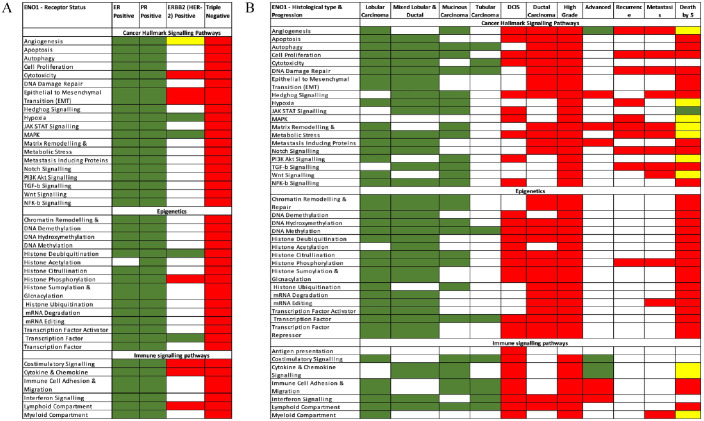
Oncomine network analysis. Network analysis of the main cancer hallmarks identified that ENO1 overexpression (Red) clusters with many signalling, epigenetic, and immune pathways in (A) triple-negative BCs when classified by subtype and (B) in ductal carcinoma in situ (DCIS), ductal and advanced/high-grade disease when classified by histopathology. The opposite (ENO1 underexpression; Green) is observed for hormonal-positive BCs and lobular, mucinous, tubular carcinomas. BC cohorts and number of cases from Oncomine are given in Supplementary Table 2.

Notably, 9 BC patient cohorts had data available on the Oncomine platform (Supplementary Table 2). Network analysis of the main cancer hallmarks and their associated pathways identified that ENO1 clusters with many signalling, epigenetic, and immune pathways. Analysis by receptor status revealed ENO1 overexpression in the triple-negative subtype for all the analysed pathways. In the HER2-positive, overexpression was linked to cytotoxicity, epithelial-to-mesenchymal transition (EMT), costimulatory, and cytokine/chemokine signalling, histone phosphorylation, whereas underexpression was linked to hypoxia, mitogen-activated protein kinase (MAPK) signalling, histone deubiquitination, and transcription factor. The hormone receptor-positive subtypes showed ENO1-associated underexpression of all the analysed pathways. All the data are summarised in Figure 6A and Supplementary Table 3.

Analysis by histological subtypes and disease progression show that ENO1 was underexpressed in the tumours with better prognosis (Figure 6B). In the lobular and mixed lobular and ductal carcinomas, ENO1 was underexpressed for almost all signalling, immune, and epigenetic pathways. It was also underexpressed in the rare mucinous and tubular carcinomas in several pathways, including autophagy, DNA damage repair, DNA methylation, transcription factor, costimulatory signalling, lymphoid compartment, and immune cell adhesion and migration (Figure 6B).

In the more aggressive ductal carcinoma, ENO1 is overexpressed and clusters with angiogenesis, apoptosis, cell proliferation, cytotoxicity, several signalling, and epigenetic pathways in the early-stage ductal carcinoma in situ (DCIS), whereas more pathways are recruited in ductal carcinoma and high-grade tumours. Interestingly, ENO1 shows clustering with all the major immune pathways in DCIS and high-grade tumours but only with interferon signalling and lymphoid compartment in ductal carcinoma. There is a general pattern for the clustering to occur in the context of ENO1 gene overexpression as the disease progresses to high-grade, advanced, recurrence, and metastasis with pathways recruited or disappearing, highlighting the importance of ENO1 in the signalling, epigenetic, and immune pathways at the different stages. A mixed profile is observed with ENO1 clustering and survival (death by 5 years) where underexpression clusters with JAK–STAT signalling and overexpression with all the epigenetic pathways, immune cell adhesion and migration, Notch, TGF-b, NFK-b, hedgehog signalling, and metastasis-inducing proteins among others. All the data according to histological types and progression are summarised in Figure 6A and Supplementary Table 4.

**Figure 7.**
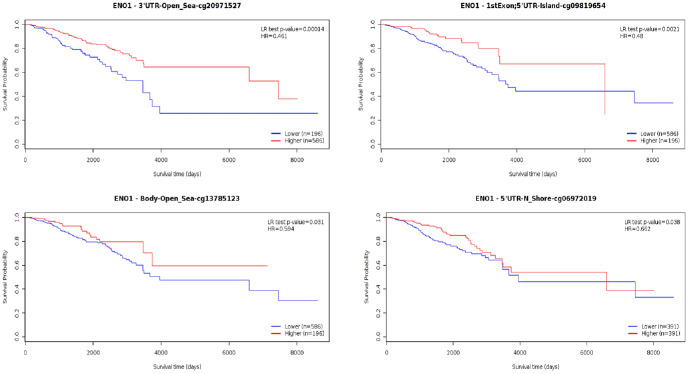
ENO1 DNA methylation and survival. Survival effect of ENO1 methylation on BC patients identified 4 loci where low methylation is linked to worse survival outcome (cg20971527: *P* = .00014; cg09819654: *P* = .0021; cg06972019: *P* = .038; cg13785123: *P* = .031).

MethSurv (https://biit.cs.ut.ee/methsurv) using data from Breast Cancers obtained from the Cancer Genome Atlas (BRCA-TCGA) (n = 782) was used to access ENO1 DNA methylation (Figure 7A) and its effect on survival.^36^ However, 4 loci were identified where low methylation is linked to worse survival outcome (Figure 7B; cg20971527: HR 0.461, CI 0.312–0.681, LR-test *P* = .00014; cg09819654: HR 0.48, CI 0.291–0.792, LR-test *P* = .0021; cg06972019: HR 0.662, CI 0.446–0.981, LR-test *P* = .038; cg13785123: HR 0.594, CI 0.36–0.978, LR-test *P* = .031). All the data are presented in Supplementary Table 5.

### Immune markers

A low-to-moderate positive correlation was observed between ENO1 expression and certain tumour-infiltrating lymphocytes (TILs), immunomodulators, and chemokines, whereas a negative correlation was observed with ENO1 methylation. ENO1 CNAs had no effect on the immune markers analysed in TISIDB. In the TILs, the strongest correlations with ENO1 expression were observed with activated dendritic cells (Act-DCs), Gamma Delta T cell (Tγδ), CD56 Natural Killer (NK) cells, and activated CD4T cells (Act_CD4). Methylation also showed a strong negative correlation with NKT cells, Act_DCs, Tγδ, T-helper 1 (Th1) and T-helper 2 (Th2) cells, Act_CD4, and activated B cells (Act_B).

A low-to-moderate positive correlation was identified between the expression of ENO1 and expression of immunoinhibitory genes, such as IDO1, IL10RB, LAG3, except PVRL2 that showed a negative correlation to ENO1 expression. Methylation of ENO1 was negatively associated with IDO1, BTLA, CTLA4, IL10, IL10RB, LAG3, and TIGIT but positively correlated to PDCD1LG2 (PDL2) and PVRL2. Similar profile was observed with most of the chemokines that negatively correlated with ENO1 methylation and to a lesser extend show a positive correlation to ENO1 expression. The correlation data are analytically presented in Supplementary Table 6, and examples of the correlation plot of immune markers and ENO1 expression/methylation are presented in Supplementary Figure 4.

**Figure 8.**
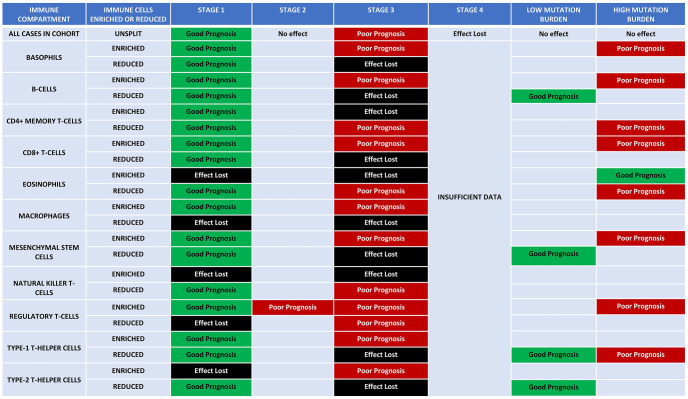
Visual depiction of the clustering of ENO1 to different immune compartments. The table highlights the differences in the immune compartments (enriched or depleted) and the effect of ENO1 at different stages of the disease and with high or low mutation burden. Green and red highlight the good and bad prognostic effect of ENO1, respectively, whereas the lighter shades show the trend. Black highlights the lost effect.

When prognostic data were split by enrichment or depletion of immune compartments, the good prognostic effect of ENO1 in Stage 1 disease was found to be dependent on enrichment of eosinophils, NKT cells, and type-2 T-helper (Th2) cells, and depletion of macrophages. The poor prognostic effect of ENO1 at Stage 3 was dependent on enrichment of CD4+ memory T cells, NKT cells and eosinophils and depletion of basophils, B cells, CD8+ T cells, macrophages, mesenchymal stem cells and both type-1T-helper (Th1) and Th2 cells (Figure 8). Similarly, enrichment of basophils, B cells, CD8+ T cells, regulatory T cells and depletion of eosinophils, CD4+ memory T cells. and Th1/Th2 in association with high mutation burden conferred poor prognosis (Figure 8).

## Discussion

This study has revealed that ENO1 is a poor prognostic marker at RNA level, particularly in triple-negative BC. The poor prognostic effect is observed in advanced (Stage 3) disease and is present in HER2-positive and triple-negative BCs. Our in silico RNA data agree with previous in vivo and in vitro studies showing an association of high ENO1 expression with the aggressive basal subtype and a favourable prognosis in patients with early-stage BC but not with advanced stage disease and basal BC.^16,26,38,39^ There was no effect observed at protein level possibly due to the low number of samples or additional modifications. Many biomarkers that are significant at RNA level are not confirmed to be predictive and prognostic at protein level due to post-transcriptional or post-translational modifications (acetylation, methylation, phosphorylation, and citrullination) affecting protein expression.^40-43^ We also observed that methylation of the gene is associated with a good prognostic effect, consistent with the expected effect from reducing ENO1 gene expression. In addition to methylation, the gene clusters with a wide range of epigenetic pathways, and this effect is established at the in situ stage.

Although genomic alterations of ENO1 in BC are rare, an increased rate of ENO1 alterations in metastatic tumours as compared with primaries was identified, indicating that ENO1 alterations accumulate with progression and treatment. Both amplifications and deletions were detected, and both are associated with a poor prognosis, providing a partial explanation for the emergence of the poor prognostic effect in more advanced disease. ENO1 clusters with overexpression of all the pathways involved in cancer hallmarks and epigenetic regulation in triple-negative breast carcinomas. We observed a tendency to recruit these pathways in the context of high-grade disease, ductal carcinoma, and tumours with a poor 5-year survival. By contrast, ENO1 is a good prognostic marker in Stage 1 disease and clusters in the context of underexpression with a wide range of the pathways in histological subtypes of BC known to be associated with a good prognosis, such as mucinous and tubular carcinomas. Clustering of ENO1 across the pathways we examined was also observed in DCIS, indicating that the gene plays a role from the earliest stages of BC development.

A complex relationship of ENO1 with immune pathways was revealed by this study to complement other studies evaluating the role of ENO1 in the tumour–immune microenvironment (TME).^13,38,39^ Underexpression occurs in good prognostic subtypes of breast carcinoma, whereas overexpression of the pathways occurs in triple-negative breast carcinomas. Overexpression of the gene tended to promote immune inhibitors and suppress chemokines. However, despite this, higher expression of the gene was correlated with increased infiltration of TILs, whereas methylation of the gene was associated with decreased infiltration of TILs, likely reflecting the dominating influence of the broader immune pathway recruitment observed in the network analysis. There will likely be functional effects of ENO1 overexpression, such as a reduced pH of the microenvironment caused by increased glycolysis, which would also tend to promote immune cell recruitment. Splitting survival data by stage and immune compartment revealed complex effects, with the poor prognostic effect at Stage 3 being dependent on enrichment or depletion of a wider range of immune cells than the good prognostic effect seen in Stage 1. The Stage 1 good prognostic effect tended to be dependent on depletion of a narrow range of immune pathways, whereas the poor prognostic effect at Stage 3 was dependent on enrichment of a wider range of different immune pathways, likely reflecting the ability of the tumour to co-opt the immune system. These novel data overall point to a potential role of ENO1 in recruiting immune pathways, particularly in triple-negative breast carcinomas and advanced disease, immediately suggesting that there may be a role for ENO1-targeted therapy as an adjunct to immune therapy. Several studies using ENO1-targeting DNA vaccines in mouse models of pancreatic ductal adenocarcinoma have shown prophylactic and therapeutic potential by inducing complement-dependent cytotoxicity, enhancing CD4 anti-tumour activity and immune cell response.^44,45^ A recent study showed that an ENO1-targeting antibody targets multiple TME niches involved in prostate cancer progression and bone metastasis via a plasmin-related mechanism reinforcing the potential use of ENO1 targeted therapies in combination to immunotherapies.^46^

The main limitation of this study is that our analysis is performed using available databases and platforms, and lacks experimental data. Nevertheless, it shows that ENO1 is overexpressed in comparison to normal tissue and confers a worse prognosis in BC supporting previous studies using cell lines and BC patient material.^16^,^23-26,47^ It also provides further insight into the ENO1 expression in relation to immune infiltration and methylation.^38,39^

## Conclusions

In summary, this novel large scope in silico analysis highlights further the potential of ENO1 as a novel biomarker of BC progression, particularly in the triple-negative/basal subtype, a subtype with worse prognosis than the hormone-positive and the HER2-positive subtypes. In addition, broad epigenetic associations are established by the time DCIS has become invasive and more are observed as the cancer progresses up to death, whereas more pathways involved in the cancer hallmarks and the immune pathways are recruited. The observations in this study warrant further investigation using patient-derived organoids, patient-derived xenografts, and mouse models of BC to characterise better the relationship of ENO1 with epigenetic and immune-related pathways. This will help the development of therapeutics targeting ENO1 directly and as an adjunctive treatment to immunotherapies.

## Supplemental Material

sj-jpg-4-bcb-10.1177_11782234241285648 – Supplemental material for ENO1 as a Biomarker of Breast Cancer Progression and Metastasis: A Bioinformatic Approach Using Available Databases Supplemental material, sj-jpg-4-bcb-10.1177_11782234241285648 for ENO1 as a Biomarker of Breast Cancer Progression and Metastasis: A Bioinformatic Approach Using Available Databases by Athina Giannoudis, Alistair Heath and Vijay Sharma in Breast Cancer: Basic and Clinical Research

sj-jpg-5-bcb-10.1177_11782234241285648 – Supplemental material for ENO1 as a Biomarker of Breast Cancer Progression and Metastasis: A Bioinformatic Approach Using Available Databases Supplemental material, sj-jpg-5-bcb-10.1177_11782234241285648 for ENO1 as a Biomarker of Breast Cancer Progression and Metastasis: A Bioinformatic Approach Using Available Databases by Athina Giannoudis, Alistair Heath and Vijay Sharma in Breast Cancer: Basic and Clinical Research

sj-jpg-6-bcb-10.1177_11782234241285648 – Supplemental material for ENO1 as a Biomarker of Breast Cancer Progression and Metastasis: A Bioinformatic Approach Using Available Databases Supplemental material, sj-jpg-6-bcb-10.1177_11782234241285648 for ENO1 as a Biomarker of Breast Cancer Progression and Metastasis: A Bioinformatic Approach Using Available Databases by Athina Giannoudis, Alistair Heath and Vijay Sharma in Breast Cancer: Basic and Clinical Research

sj-jpg-7-bcb-10.1177_11782234241285648 – Supplemental material for ENO1 as a Biomarker of Breast Cancer Progression and Metastasis: A Bioinformatic Approach Using Available Databases Supplemental material, sj-jpg-7-bcb-10.1177_11782234241285648 for ENO1 as a Biomarker of Breast Cancer Progression and Metastasis: A Bioinformatic Approach Using Available Databases by Athina Giannoudis, Alistair Heath and Vijay Sharma in Breast Cancer: Basic and Clinical Research

sj-xlsx-1-bcb-10.1177_11782234241285648 – Supplemental material for ENO1 as a Biomarker of Breast Cancer Progression and Metastasis: A Bioinformatic Approach Using Available Databases Supplemental material, sj-xlsx-1-bcb-10.1177_11782234241285648 for ENO1 as a Biomarker of Breast Cancer Progression and Metastasis: A Bioinformatic Approach Using Available Databases by Athina Giannoudis, Alistair Heath and Vijay Sharma in Breast Cancer: Basic and Clinical Research

sj-xlsx-2-bcb-10.1177_11782234241285648 – Supplemental material for ENO1 as a Biomarker of Breast Cancer Progression and Metastasis: A Bioinformatic Approach Using Available Databases Supplemental material, sj-xlsx-2-bcb-10.1177_11782234241285648 for ENO1 as a Biomarker of Breast Cancer Progression and Metastasis: A Bioinformatic Approach Using Available Databases by Athina Giannoudis, Alistair Heath and Vijay Sharma in Breast Cancer: Basic and Clinical Research

sj-xlsx-3-bcb-10.1177_11782234241285648 – Supplemental material for ENO1 as a Biomarker of Breast Cancer Progression and Metastasis: A Bioinformatic Approach Using Available Databases Supplemental material, sj-xlsx-3-bcb-10.1177_11782234241285648 for ENO1 as a Biomarker of Breast Cancer Progression and Metastasis: A Bioinformatic Approach Using Available Databases by Athina Giannoudis, Alistair Heath and Vijay Sharma in Breast Cancer: Basic and Clinical Research

## Declarations

**Ethics approval and consent to participate:** Not applicable

**Consent for publication:** Not applicable

**Availability of data and materials:** All data generated or analysed during this study are included in this article and its supplementary information files. The datasets supporting the conclusions of this article have been generated using the following websites: www.kmplotter.com, http://www.g-2-o.com; http://www.cbioportal.org; https://www.oncomine.org; https://biit.cs.ut.ee/methsurv/; www.rocplotter.com; https://epifactors.autosome.ru; http://cis.hku.hk/TISIDB/; and appropriate references to these are included in the article.
