# Supplementary figures and images for: ENO1 as a Biomarker of Breast Cancer Progression and Metastasis: A Bioinformatic Approach Using Available Databases
Source: Breast Cancer (Auckl). 2024 Oct 19;18:11782234241285648. doi: 10.1177/11782234241285648 (PMC11526306; doi:10.1177/11782234241285648)

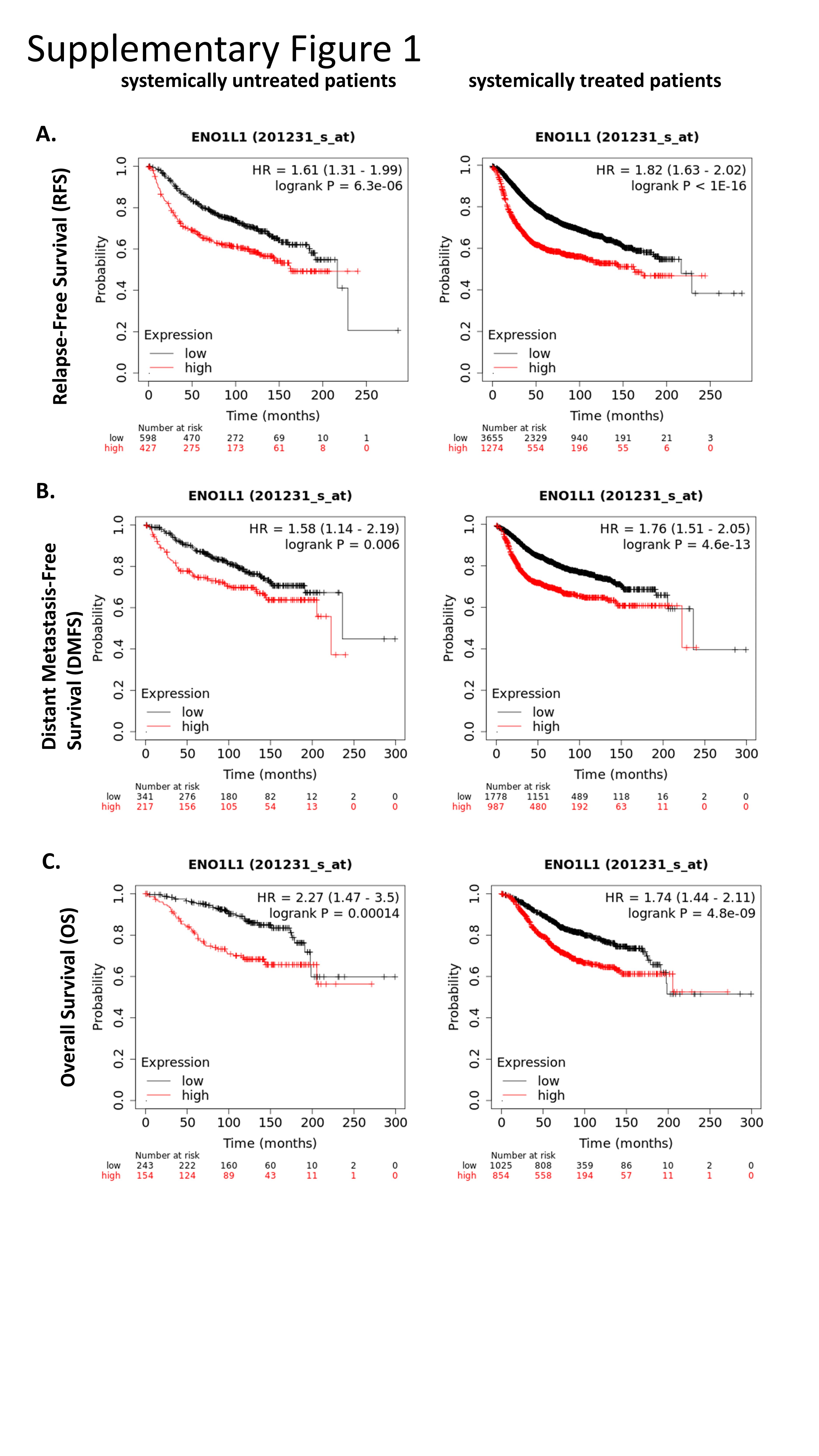

Supplement: sj-jpg-4-bcb-10.1177_11782234241285648 – Supplemental material for ENO1 as a Biomarker of Breast Cancer Progression and Metastasis: A Bioinformatic Approach Using Available Databases [file sj-jpg-4-bcb-10.1177_11782234241285648.jpg]

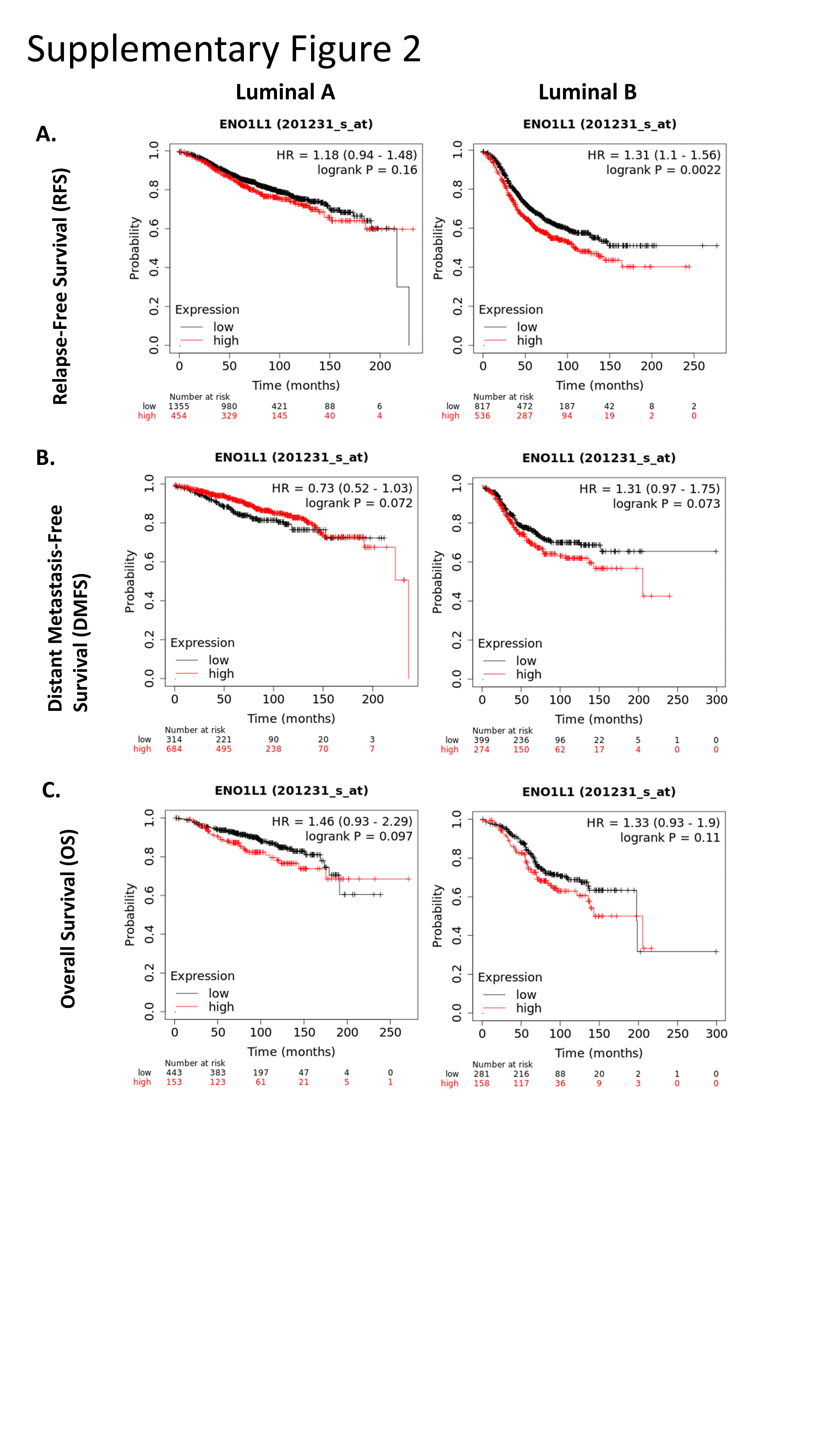

Supplement: sj-jpg-5-bcb-10.1177_11782234241285648 – Supplemental material for ENO1 as a Biomarker of Breast Cancer Progression and Metastasis: A Bioinformatic Approach Using Available Databases [file sj-jpg-5-bcb-10.1177_11782234241285648.jpg]

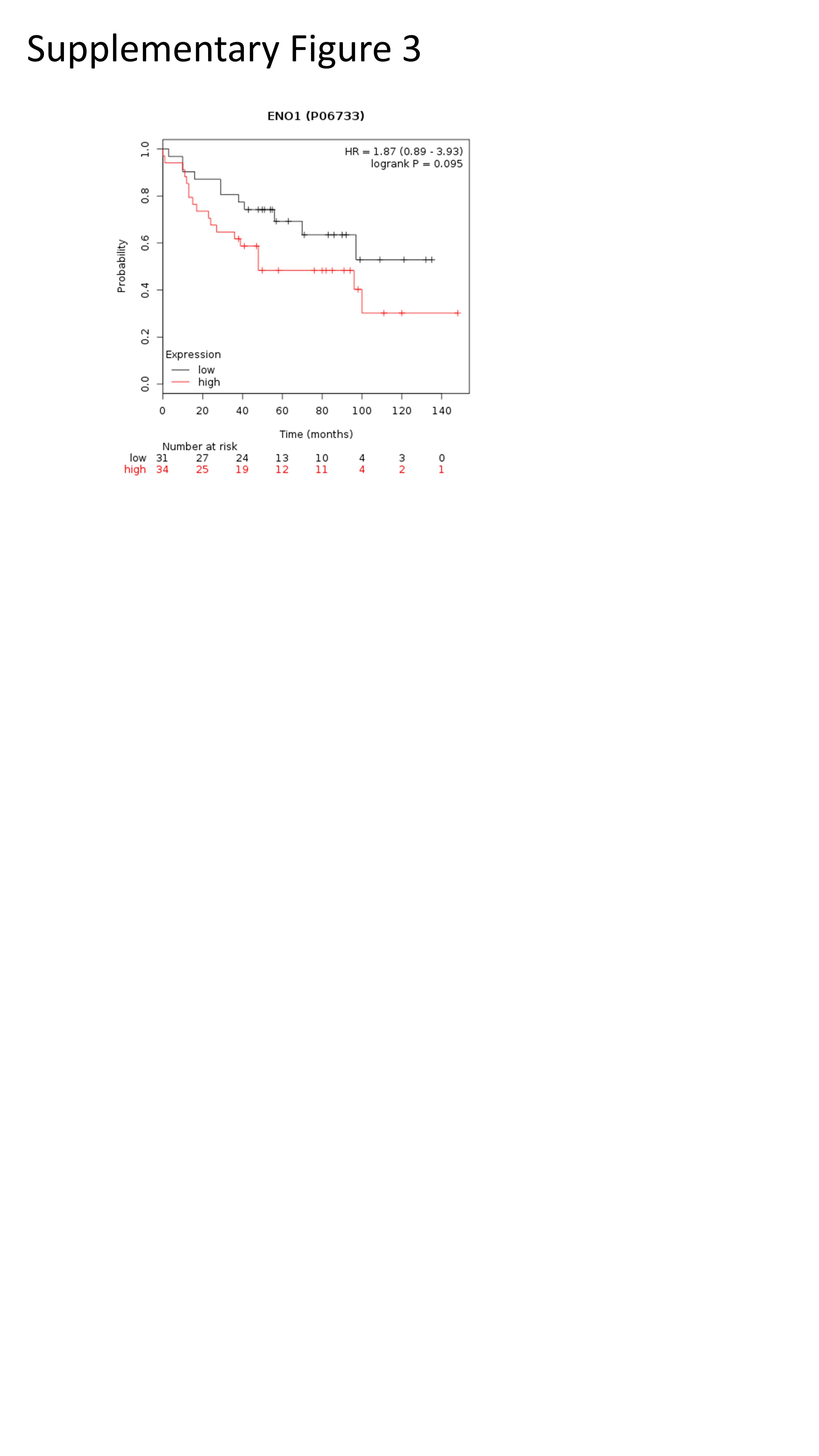

Supplement: sj-jpg-6-bcb-10.1177_11782234241285648 – Supplemental material for ENO1 as a Biomarker of Breast Cancer Progression and Metastasis: A Bioinformatic Approach Using Available Databases [file sj-jpg-6-bcb-10.1177_11782234241285648.jpg]

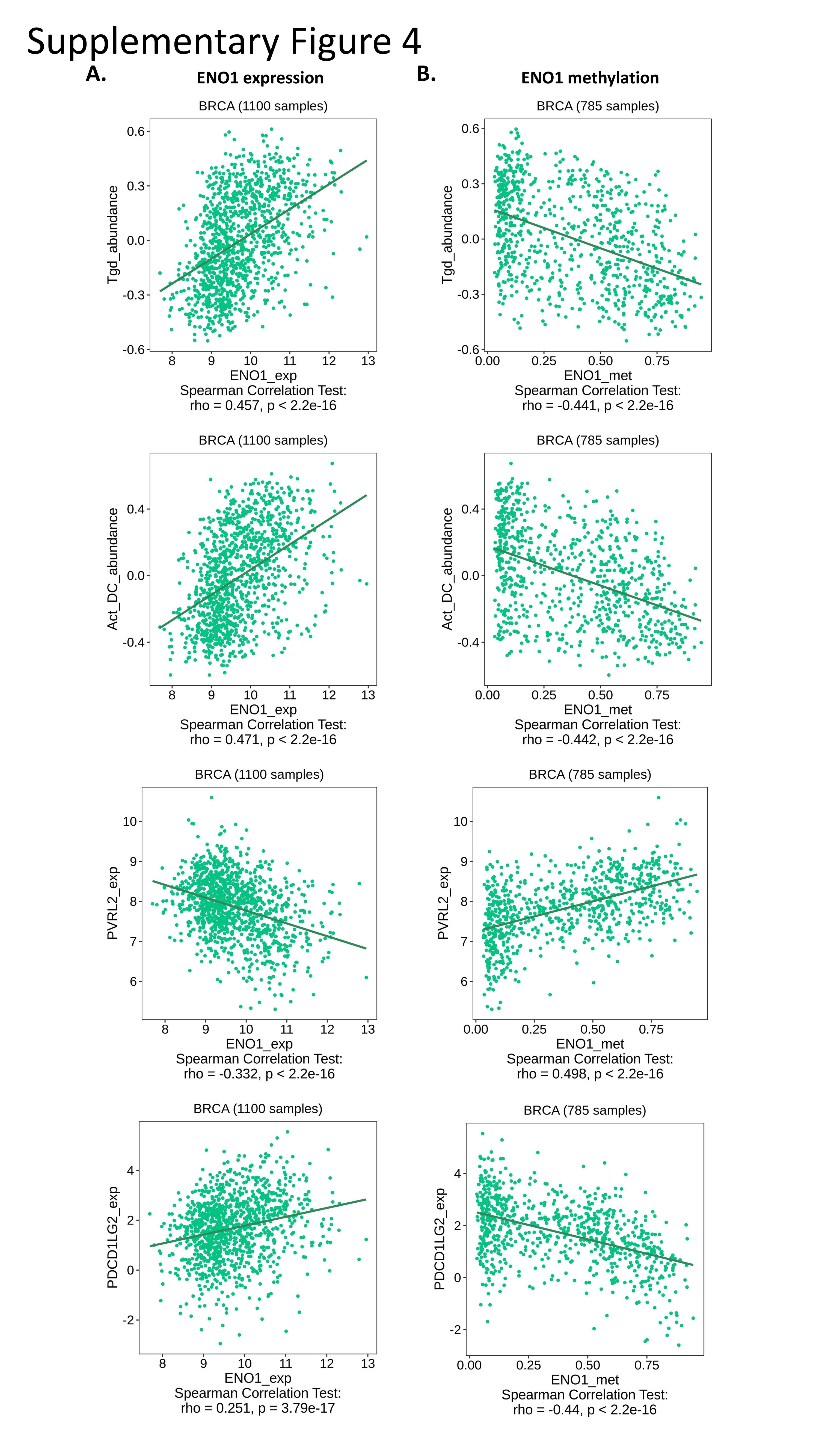

Supplement: sj-jpg-7-bcb-10.1177_11782234241285648 – Supplemental material for ENO1 as a Biomarker of Breast Cancer Progression and Metastasis: A Bioinformatic Approach Using Available Databases [file sj-jpg-7-bcb-10.1177_11782234241285648.jpg]
